# The impact of acute rosiglitazone on insulin pharmacokinetics at the blood‐brain barrier

**DOI:** 10.1002/edm2.149

**Published:** 2020-06-04

**Authors:** Demi C. Galindo, William A. Banks, Elizabeth M. Rhea

**Affiliations:** ^1^ Department of Medicine University of Washington Seattle WA USA; ^2^ Research and Development Veterans Affairs Puget Sound Healthcare System Seattle WA USA

**Keywords:** blood‐brain barrier, insulin, pharmacokinetics, rosiglitazone

## Abstract

**Introduction:**

CNS insulin levels are decreased and insulin receptor signalling is dampened in Alzheimer's disease (AD). Increasing CNS insulin levels through a variety of methods has been shown to improve memory. Indeed, medications routinely used to improve insulin resistance in type 2 diabetes are now being repurposed for memory enhancement. CNS insulin is primarily derived from the circulation, by an active transport system at the blood‐brain barrier (BBB). The goal of this study was to determine whether rosiglitazone (RSG), a drug used to improve insulin sensitivity in type 2 diabetes, could enhance insulin transport at the BBB, as a potential therapeutic for improving memory.

**Methods:**

Using radioactively labelled insulin and the multiple‐time regression analysis technique, we measured the rate of insulin BBB transport and level of vascular binding in mice pretreated with vehicle or 10 µg RSG in the presence or absence of an insulin receptor inhibitor.

**Results:**

Although we found acute RSG administration does not affect insulin transport at the BBB, it does restore BBB vascular binding of insulin in an insulin receptor–resistant state.

**Conclusions:**

Acute RSG treatment does not alter insulin BBB transport in healthy mice but can restore insulin receptor binding at the BBB in an insulin‐resistant state.

## Graphical abstract

Here, the impact of rosiglitazone (RSG) on insulin transport across the murine BBB, the primary means for CNS access to insulin, is investigated. While RSG did not affect the transport rate of insulin across the BBB, it did improve vascular binding under conditions in which the insulin receptor was acutely inhibited.

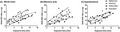

## 1 INTRODUCTION

As people with type 2 diabetes, a disease associated with peripheral insulin resistance, have nearly a two‐fold increase in developing Alzheimer's disease (AD),^1^ a disease associated with central insulin resistance, it is important to understand the potential relationship between these two types of insulin resistance. Reduced insulin levels and insulin resistance in the brain have been associated with AD since the late 1990s.^2^, ^3^, ^4^, ^5^ Although peripheral insulin resistance can be tested by various measures relating to glucose uptake, central nervous system (CNS) insulin resistance, as defined by impaired insulin action in the brain, is more difficult to determine, part of which is due to the limited role in insulin mediating CNS glucose uptake at the blood‐brain barrier (BBB).^6^ A ratio of cerebrospinal fluid to serum insulin levels can help indicate CNS insulin resistance.^3^ However, most studies have evaluated CNS insulin resistance in post‐mortem human brain slices, measuring the response to ex vivo insulin stimulation and subsequent downstream insulin signalling.^7^, ^8^ As the focus of the work presented here is at the BBB, rather than the CNS or peripheral organs, insulin resistance in this study is described by the loss of insulin binding to the insulin receptor.

Endogenous CNS insulin is primarily derived from the periphery by a saturable transport system located at the BBB.^9^, ^10^ If CNS insulin resistance occurs due to impaired access from the BBB, improving transport across the BBB could improve CNS insulin resistance,^11^ similar to what has been proposed for CNS leptin resistance and the development of obesity.^12^, ^13^ Insulin in the CNS can act both as a trophic factor as well as a growth factor, regulating cell growth, mitochondrial function, synaptic plasticity and cognitive function. Therefore, alterations of insulin transport across the BBB have the potential to affect memory. Indeed, increasing the central nervous system (CNS) insulin level has been proven to be beneficial by improving memory.^14^, ^15^, ^16^

Several pharmacologic options are used to treat peripheral insulin resistance and restore insulin receptor sensitivity, such as those used in type 2 diabetes. These drugs are now being repurposed for use in memory improvement. Antidiabetic drugs that activate peroxisome proliferator‐activated receptor gamma (PPARγ) improve cognition in diabetic mice,^17^ in the aged rat^18^ and in AD mice.^19^, ^20^ However, the clinical data regarding the effect of PPARγ activation on memory improvement have been mixed. While some studies have shown improvements,^21^, ^22^, ^23^ others have been negative.^24^, ^25^, ^26^ Rosiglitazone (RSG) is a PPARγ agonist used to enhance insulin sensitivity via various mechanisms.^27^ RSG can decrease phosphorylation of proteins that would otherwise dysregulate insulin receptor activated pathways.^27^ Perhaps the differences in beneficial effect of RSG in clinical and preclinical studies are due to the ability of RSG to access the CNS. Indeed, RSG is able to cross the BBB in mice but it is also a ligand for P‐glycoprotein.^28^ In human brain endothelial cells, the brain‐to‐blood transport of RSG is two times greater than the blood‐to‐brain influx.^28^ Therefore, it is possible in humans that RSG is unable to sufficiently access the CNS. Despite the link between type 2 diabetes and CNS insulin resistance, the effect of PPARγ agonists at the BBB on insulin transport have not been explored.

In the current study, our research explores the effects of RSG on insulin pharmacokinetics at the murine BBB to measure the transport rate of insulin as well as the binding capacity of insulin to the brain endothelium. Further, we used the selective antagonist to the insulin receptor, S961,^29^ to determine what effects RSG might have in an insulin‐resistant state. Acute treatment with S961 has been previously shown to induce hyperglycaemia and glucose intolerance.^30^ We focus on regions within the whole brain that are well documented for their role in CNS insulin signalling: the olfactory bulb has the fastest transport rate for insulin across the BBB^31^ and is an important memory centre in the rodent, the hippocampus is important in memory, and the hypothalamus plays a role in peripheral metabolism.

## 2 MATERIALS AND METHODS

### 2.1 Animals

Eight‐week‐old male CD‐1 mice were purchased from Charles River Laboratories. CD‐1 mice are an established model for BBB transport studies.^9^, ^32^, ^33^ Mice had free access to food and water and were maintained on a 12‐hour dark (18:00‐06:00 hour)/12‐hour light (06:00‐18:00 hour) cycle in a room with a controlled temperature (24 ± 1°C) and humidity (55 ± 5%). All studies were approved by the certified local Animal Care and Use Committee and were performed in a facility approved by the Association for Assessment and Accreditation of Laboratory Animal Care. For all studies, mice were anaesthetized with a 0.15 mL intraperitoneal injection of 40% urethane at the beginning of each study to minimize pain and distress.

### 2.2 Radioactive labeling

Ten micrograms of human insulin (Sigma‐Aldrich) were radioactively labelled by the chloramine‐T (Sigma‐Aldrich) method with 1 mCi ^125^I (Perkin Elmer) as previously described.^34^ Radiolabelled insulin was separated from free iodine on a Sephadex G‐10 column (Sigma‐Aldrich). ^125^I‐insulin was prepared on the day prior or day of experiment. The specific activity of ^125^I‐insulin is 55 Ci/g as previously calculated.^9^

One milligram of bovine serum albumin (Sigma‐Aldrich) was radioactively labelled via stannous tartrate (Fisher, MP Biomedicals) with 1 mCi ^99m^Tc (GE Healthcare).^34^ Radiolabelled albumin was separated from free ^99m^Tc on a Sephadex G‐10 column. This agent has a half‐life of 6 hours and therefore was prepared freshly on the day of each experiment. Radioactivity was consistently over 90% in the 15% trichloroacetic acid precipitated fractions for both insulin and albumin to confirm successful radioactive labelling.

### 2.3 Pretreatment with RSG

All mice were pretreated with a 0.2 mL iv injection of 10% dimethyl sulfoxide (DMSO) in lactated Ringer's solution (LR) (DMSO vehicle) or 10 µg rosiglitazone maleate (RSG; Cayman Chemicals, Ann Arbor, MI) in 10% DMSO/LR thirty minutes prior to measurement of ^125^I‐insulin BBB transport by either the blood‐to‐brain transport method or transcardiac brain perfusion method. This experimental set‐up was based on previous studies investigating the acute effect of rosiglitazone.^35^, ^36^

### 2.4 Blood‐to‐brain ^125^I‐insulin transport

Following pretreatment with DMSO or RSG, a second 0.1 mL jugular vein iv injection containing 1 × 10^6^ counts per minute (cpm) ^125^I‐insulin and 5 × 10^5^ cpm ^99m^Tc‐albumin in 1% bovine serum albumin (BSA)/LR ± 1 µg S961 (Novo Nordisk) was administered. ^99m^Tc‐albumin was co‐injected as a marker for vascular space.^37^ At 0.5‐10 minutes after the injection, blood was collected from the carotid artery. Mice were decapitated, and the brain was collected immediately, dissected into the hypothalamus, olfactory bulb and remaining whole brain and weighed. Blood was centrifuged at 5400*g* for 10 min and serum collected. Radioactivity in 50 µL of serum and entire brain samples were counted in a gamma counter (Wizard2; Perkin Elmer). The brain/serum (B/S) ratio (μL/g) of ^125^I‐insulin and ^99m^Tc‐albumin in each gram of brain sample was calculated separately.

### 2.5 Transcardiac brain perfusion

Following pretreatment with DMSO or RSG, the thorax was opened, heart exposed, both jugulars severed and the descending thoracic aorta clamped. A 26‐gauge butterfly needle was inserted into the left ventricle of the heart, and Zlokovic's buffer (7.19 g/L NaCl, 0.3 g/L KCl, 0.28 g/L CaCl_2_, 2.1 g/L NaHCO_3_, 0.16 g/L KH_2_PO_4_, 0.17 g/L anhydrous MgCl_2_, 0.99 g/L D‐glucose and 1% BSA) containing 2 × 10^5^ cpm ^125^I‐insulin was infused at a rate of 2 mL/min for 1‐5 minutes. The perfusate was freshly prepared for each study day. Perfusate was collected throughout the study to determine the average cpm/µL of perfusate. After perfusion, the olfactory bulb was collected and the brain dissected into regions (frontal/parietal/occipital cortex, striatum, hypothalamus, hippocampus, thalamus, cerebellum, midbrain and pons/medulla) according to Glowinski and Iversen;^38^ each region was weighed separately. The amount of radioactivity was determined by a gamma counter (Wizard2, Perkin Elmer). Brain/perfusate ratios are calculated by dividing the cpm in a gram of brain by the cpm in a µL of perfusate to yield units of µL/g. In order to determine whether the 10% DMSO solution was affecting ^125^I‐insulin transport, we also pre‐injected another set of mice with 1% BSA/LR prior to transcardiac brain perfusion.

### 2.6 Multiple‐time regression analysis

Multiple‐time regression analysis was used as detailed previously^37^, ^39^, ^40^ to calculate the rate of unidirectional influx for ^125^I‐insulin. For blood‐to‐brain transport studies, the B/S ratios for ^125^I‐insulin are corrected for vascular space by subtracting the corresponding ratio for ^99m^Tc‐albumin, yielding a delta B/S ratio. Exposure time is calculated by the formula:

(1) $$\text{Exposure time}\;=\;\frac{\int_{0}^{t}\text{Cp}(t)dt}{\text{Cp}(t)}$$

where Cp*(t)* is the level of radioactivity (cpm) in serum at time (*t*). Exposure time corrects for the clearance of peptide from the blood. For blood‐to‐brain studies, the B/S ratios are plotted against the exposure time to calculate the influx of ^125^I‐insulin using the same formula originally described in Equation 6 by Blasberg et al,^37^ updated by Kastin et al using GraphPad^40^:

(2) $$\frac{\text{Am}}{\text{Cp}t}=Ki\left(\frac{\int_{0}^{t}\text{Cp}(t)dt}{\text{Cp}(t)}\right)+V_{i}$$

where Am is level of radioactivity (cpm) per g of brain tissue at time *t*, Cp*t* is the level of radioactivity (cpm) per μL serum at time *t*, *K*_i_ (μL/g‐min) is the unidirectional solute influx from blood to brain and *V*_i_ (μL/g) is the level of rapid and reversible binding for the brain vasculature. The slope of the linearity measures *K*_i_ and is reported with its standard error term. The y‐intercept of the linearity measures *V*_i_, the initial volume of distribution in brain at *t* = 0.^37^ Blasberg, Fenstermacher and Patlak originally described *V*_i_ as the volume of test substance that rapidly and reversibly exchanges with the plasma (Blasberg 1983). For cardiac perfusion studies, formula 2 is employed, using the brain/perfusate ratios and the clock time is used in place of exposure time.

### 2.7 Capillary depletion in mice

To determine whether RSG altered brain capillary sequestration of ^125^I‐insulin, we performed capillary depletion as adapted to mice.^41^, ^42^ Following pretreatment with DMSO or RSG, a second 0.1 mL jugular vein iv injection containing 1 × 10^6^ counts per minute (cpm) ^125^I‐insulin and 5x10^5^ cpm ^99m^Tc‐albumin in 1% BSA/LR. Two and a half minutes later, blood was collected from the carotid, the mice decapitated and the whole brain removed. The brain was homogenized with ten strokes of a glass homogenizer in 0.8 mL of physiological buffer (10 mmol/L HEPES, 141 mmol/L NaCl, 4 mmol/L KCl, 2.8 mmol/L CaCl_2_, 1 mmol/L MgSO_4_, 1 mmol/L NaH_2_PO_4_ and 10 mmol/L D‐glucose adjusted to pH 7.4). A 26% dextran solution in the same physiological buffer (1.6 mL) was added to the homogenate and vortexed, and the solution was homogenized with three more strokes. Homogenates were then centrifuged at 3500 *g* for 20 minutes at 4°C with a swinging bucket rotor (Beckman Coulter). The brain capillary fraction contained in the pellet and the supernatant containing the brain parenchyma were carefully separated. Fractions and 50 µL serum were counted in a gamma counter (Wizard2, Perkin Elmer). The B/S ratio of radioactivity of ^125^I‐insulin in the supernatant (parenchyma) was corrected for vascular space by subtracting the B/S ratio of ^99m^Tc‐albumin in the supernatant. Fractions were expressed as percentages of total brain.

### 2.8 Data analysis

Regression analyses and other statistical analyses were performed with the use of Prism 8.0 (GraphPad Software Inc). For pharmacokinetic studies (multiple‐time regression analysis), the slope of the linear regression lines (*K*_i_), reported with their correlation coefficients (*r*), and y‐intercepts (*V*_i_) were compared statistically with the Prism 8.0 software package. Differences in *V*_i_ were compared by two‐way analysis of variance (ANOVA) followed by Sidak's post hoc test to determine differences due to RSG and S961 treatment.

## 3 RESULTS

### 3.1 Serum clearance of ^125^I‐insulin following RSG treatment

**Figure 1:**
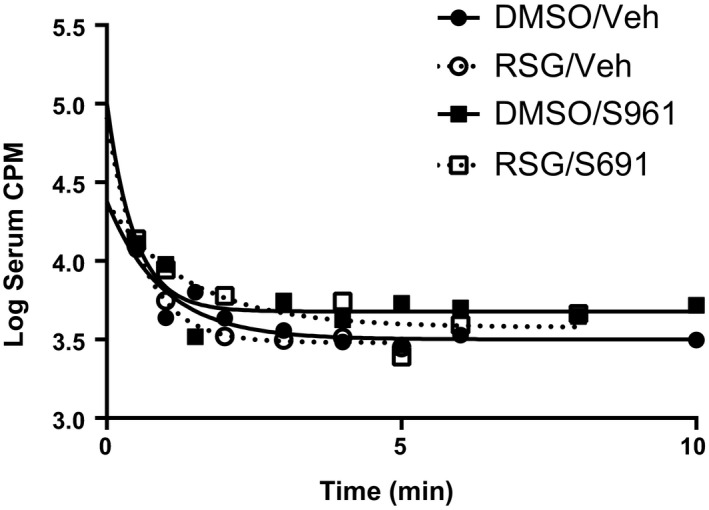
^125^I‐insulin serum clearance due to iv 30‐min pretreatment with RSG (10 µg) or cotreatment with S961 (1 µg)

Following pretreatment with DMSO or 10 µg RSG, we measured the rate of clearance from blood of ^125^I‐insulin clearance ± 1 µg S961 using a logarithmic scale. All groups had similar exponential decay curves of insulin (Figure 1). There was no effect of RSG or S961 on ^125^I‐insulin clearance. The average half‐time clearance for ^125^I‐insulin was 1.54 minutes.

### 3.2 ^125^I‐insulin BBB pharmacokinetics due to RSG: serum contribution

**Figure 2:**
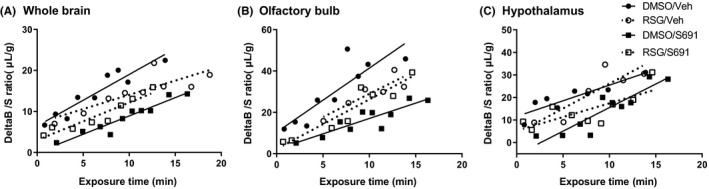
^125^I‐insulin pharmacokinetics due to iv 30‐min pretreatment with RSG (10 µg) or cotreatment with S961 (1 µg) in (A) whole brain, (B) olfactory bulb and (C) hypothalamus

Whole brain, olfactory bulb and the hypothalamus were analysed for differences in the rate of ^125^I‐insulin transport (slope, *K*_i_) and binding of ^125^I‐insulin to brain capillaries (y‐intercept, *V*_i_). Pretreatment with RSG did not significantly alter the rate of ^125^I‐insulin BBB transport in whole brain, olfactory bulb or hypothalamus (Figure 2). As shown previously,^34^ inhibition of the insulin receptor with S961 did not affect ^125^I‐insulin BBB transport in whole brain (Figure 2A). In addition, S961 did not affect ^125^I‐insulin transport in the olfactory bulb (Figure 2B) or hypothalamus (Figure 2C). Transport rates (*K*_i_) were calculated based on the linear transport data of Figure 2 and are presented in Table 1. Average rates of transport (*K*_i_) were 1.10 µL/g‐min (whole brain), 2.25 µL/g‐min (olfactory bulb) and 1.75 µL/g‐min (hypothalamus).

**Table 1:** ^125^I‐insulin BBB transport pharmacokinetics

Treatment	Whole brain			Olfactory bulb			Hypothalamus		
	*K*_i_ (µL/g‐min)	*r*	*V*_i_ (µL/g)***	*K*_i_ (µL/g‐min)	*r*	*V*_i_ (µL/g)***	*K*_i_ (µL/g‐min)	*r*	*V*_i_ (µL/g)**
DMSO/Veh	1.26 ± 0.17	.94	6.5 ± 1.3	3.10 ± 0.64	.88	10.4 ± 4.8	1.39 ± 0.28	.90	11.6 ± 2.0
RSG/Veh	1.16 ± 0.23	.93	4.1 ± 2.1	2.24 ± 0.60	.88	6.6 ± 6.2	2.21 ± 0.63	.87	4.1 ± 5.8
DMSO/S961	0.89 ± 0.09	.96	−0.008 ± 1.0	1.49 ± 0.28	.88	2.2 ± 2.9	2.09 ± 0.34	.91	−5.4 ± 3.6
RSG/S961	1.07 ± 0.09	.98	3.9 ± 0.1	2.18 ± 0.41	.91	7.1 ± 3.3	1.22 ± 0.40	.76	5.2 ± 3.4

Note

^125^I‐insulin BBB transport rate (*K*_i_), correlation coefficient (*r*) and level of vascular binding (*V*_i_) are derived from Figure 2 and expressed ± SE, n = 6‐10/group. **P < .001, ***P < .0001.

**Figure 3:**
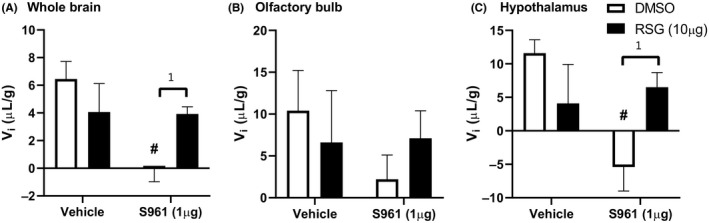
^125^I‐insulin binding to the brain vasculature (*V*_i_) due to iv 30‐min pretreatment with RSG (10 µg) or co‐treatment with S961 (1 µg) in (A) whole brain, (B) olfactory bulb and (C) hypothalamus. *P < .05 as marked; ^#^ *P* < .05 vs DMSO/VEH

The amount of vascular binding (*V*_i_) was statistically different in the whole brain and hypothalamus, but not the olfactory bulb (Figure 3). In whole brain, there was a significant interaction between RSG and S961 treatment (*P* = .015, *F* (1, 29) = 6.74). In post hoc analysis, there was a significant effect of S961 treatment (*P* = .011, F (1, 29) = 7.37), with S961 decreasing vascular binding (*P* = .0006). Pretreatment with RSG prevented this decrease in binding (*P* = .044). In the hypothalamus, there was a significant interaction between RSG and S961 treatment (*P* = .009, *F* (1, 29) = 7.90). In addition, there was a significant effect of S961 treatment (*P* = .043, *F* (1, 29) = 4.47), with S961 decreasing vascular binding (*P* = .002). Pretreatment with RSG prevented this decrease in binding (*P* = .025).

In order to verify ^125^I‐insulin was fully crossing the brain endothelial cell, we separated the brain capillary fraction from the brain parenchyma to measure the percentage of radioactivity present in each fraction. The majority of ^125^I‐insulin was present in the brain parenchyma and there were no overt differences due to RSG treatment (data not shown).

### 3.3 Effect of DMSO on ^125^I‐insulin BBB transport

**Figure 4:**
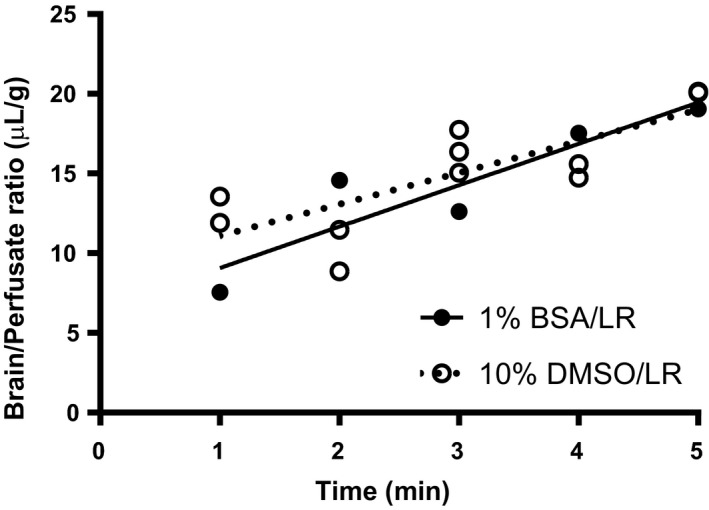
Effect of the 10% DMSO vehicle on ^125^I‐insulin pharmacokinetics compared to 1% BSA/LR in whole brain

DMSO is an organic reagent commonly used to dissolve substances. To determine if the vehicle used to dissolve RSG (10% DMSO/LR) had an effect on ^125^I‐insulin transport, we compared transport following a 30‐min pretreatment of 10% DMSO with a physiological solution, 1% BSA/LR (Figure 4). There was no effect on ^125^I‐insulin transport (*K*_i_ =2 .60 ± 0.68 µL/g‐min for 1% BSA/LR vs 1.98 ± 0.50 µL/g‐min for 10% DMSO) or vascular binding (*V*_i_ = 6.5 ± 2.3 µL/g for 1% BSA/LR vs 9.1 ± 1.7 µL/g for 10% DMSO) due to a 30‐minutes pretreatment of 10% DMSO/LR.

### 3.4 ^125^I‐insulin BBB pharmacokinetics due to RSG: absence of serum

**Figure 5:**
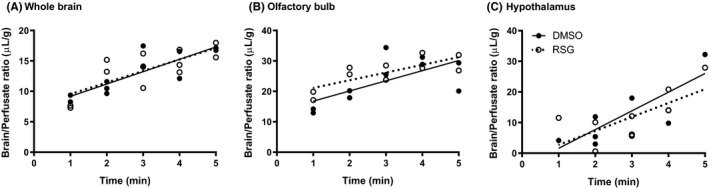
^125^I‐insulin pharmacokinetics following cardiac perfusion due to iv 30‐min pretreatment with RSG (10 µg) in (A) whole brain, (B) olfactory bulb and (C) hypothalamus

To determine if RSG could affect the BBB directly in the context of ^125^I‐insulin transport, we wanted to eliminate all serum factors and investigate the impact of RSG on ^125^I‐insulin BBB pharmacokinetics in a cardiac perfusion model, using Zlokovic's buffer as the physiological perfusate. To make sure there were not regional differences in the effect of RSG, we investigated ^125^I‐insulin transport in individual brain regions and summed up these data to derive whole‐brain transport data. There was no difference in the rate of ^125^I‐insulin transport across the BBB or changes in vascular binding due to RSG pretreatment in whole brain, olfactory bulb or hypothalamus (Figure 5). Transport rates (*K*_i_) were calculated based on the linear transport data of Figure 5 and are presented in Table 2. Of the other regions investigated (striatum, frontal/parietal/occipital cortex, hippocampus, thalamus, cerebellum, midbrain and pons/medulla), there was no effect of RSG on ^125^I‐insulin BBB pharmacokinetics (data not shown).

**Table 2:** ^125^I‐insulin BBB transport pharmacokinetics following cardiac perfusion

Treatment	Whole brain			Olfactory bulb			Hypothalamus		
	*K*_i_ (µL/g‐min)	*r*	*V*_i_ (µL/g)	*K*_i_ (µL/g‐min)	*r*	*V*_i_ (µL/g)	*K*_i_ (µL/g‐min)	*r*	*V*_i_ (µL/g)
DMSO	2.03 ± 0.49	.84	7.1 ± 1.4	3.34 ± 1.31	.67	13.5 ± 4.4	6.11 ± 1.92	.82	−4.5 ± 5.7
RSG	1.90 ± 0.50	.76	7.7 ± 1.7	2.53 ± 0.73	.77	18.6 ± 2.4	4.55 ± 1.65	.70	−1.8 ± 5.4

Note

^125^I‐insulin BBB transport rate (*K*_i_), correlation coefficient (*r*) and level of vascular binding (*V*_i_) are derived from Figure 5 and expressed ± SE, n = 7‐11/group.

## 4 DISCUSSION

Thiazolidinediones are routinely used in the treatment of type 2 diabetes to improve insulin resistance. Increasing insulin sensitivity at the receptor of insulin‐sensitive tissues increases their uptake of glucose, causing a reduction in glucose, as exemplified by the use of PPARγ agonists.^43^ There has recently been a repurposing of diabetic drugs for improvements in memory,^44^ likely due to the link between diabetes and AD.^45^ Patients with type 2 diabetes have almost double the risk for developing AD.^1^ This association between CNS and peripheral insulin resistance demonstrates the rationale for exploring insulin‐sensitizing drugs, such as those used for type 2 diabetes.^46^ Here, we investigated the impact of RSG on insulin transport across the murine BBB, the primary means for CNS access to insulin.^47^

While RSG did not affect the transport rate of insulin across the BBB, it did improve vascular binding under conditions in which the insulin receptor was acutely inhibited. In our studies, we include a marker for vascular volume, ^99m^Tc‐Alb, and can correct the amount of ^125^I‐insulin present with the amount of substrate present in the vasculature. Therefore, vascular binding (*V*_i_) in these studies reflects the amount of reversible insulin‐binding sites on the vascular endothelium, the majority of which are insulin receptors. Restoration of vascular binding could in and of itself act in an endocrine‐like manner by altering release of abluminal factors that impact the CNS.^48^

We explored the impact of RSG on insulin BBB transport both on the direct effect at the BBB as well as the impact due to blood‐borne factors. In the presence of blood factors, RSG did not affect ^125^I‐insulin transport or binding by itself. However, we also explored the effect of RSG when the insulin receptor was inhibited using the selective antagonist, S961.^29^ S961 treatment in mice and rats has been shown to induce features of type 2 diabetes including hyperglycaemia, glucose intolerance and impaired insulin sensitivity.^30^, ^49^ Similar to previous studies, there was no change in the rate of ^125^I‐insulin transport across the BBB when the receptor was inhibited, yet the amount of vascular binding for ^125^I‐insulin was decreased.^34^, ^50^ When mice were pretreated with RSG, this decrease in binding was reversed. In a separate study, pioglitazone, another PPARγ agonist, was able to reverse the effects of S961, restoring insulin sensitivity.^30^ Three potential mechanisms could explain this reversal: RSG could (a) block the binding of the inhibitor, (b) enhance the ability of the insulin receptor to bind insulin or (c) aid in increased recycling of the insulin receptor to the cell membrane to increase the amount of insulin binding. The first point could be tested by measuring the level of vascular binding of radiolabelled S961 following RSG treatment. Previous studies show S961 binds the vascular endothelium in high levels, without crossing the BBB.^34^ The second point is based on previous structural data suggesting complexity in the initial binding of insulin to the receptor.^51^, ^52^ Trafficking of the insulin receptor to the plasma membrane is not as well characterized as other receptors. However, there are proteins known to regulate expression levels of the insulin receptor at the cell surface ^53^ of which could be affected by RSG through signalling by phosphorylation of AMPK or Akt. This last mechanism could be molecularly tested by measuring changes in protein expression at the membrane surface.

Following these studies, we wanted to verify our vehicle (10% DMSO/LR) was not affecting ^125^I‐insulin transport. Following an iv 30‐minutes pretreatment of 10% DMSO/LR or a more physiological injectate solution, 1% BSA/LR, we measured ^125^I‐insulin pharmacokinetics. We did not observe any difference in ^125^I‐insulin transport or vascular binding, confirming that the vehicle the RSG is dissolved in does not impact our primary outcome.

By studying ^125^I‐insulin transport in the presence of blood, we could not deduce whether the impact of RSG was due to a change in serum factors in mediating insulin transport. Therefore, we examined the direct effects of RSG on ^125^I‐insulin brain uptake from cerebral circulation in the absence of blood‐borne factors by brain perfusion. Again, there was no difference in the transport rate or amount of vascular binding due to RSG. By eliminating any contribution of serum factors, we can conclude RSG does not have a direct effect on the BBB to alter ^125^I‐insulin pharmacokinetics in healthy mice. The transport rate and level of vascular binding for ^125^I‐insulin were similar between the two methods employed to measure pharmacokinetics (in the presence and absence of serum) in the whole brain and olfactory bulb, and similar values to previous studies reporting on the whole brain.^54^ However, in the absence of serum, the transport rate of insulin across the BBB in the hypothalamus nearly tripled and the amount of vascular binding was over 3‐fold lower (similar to the level of vascular binding when S961 is present in serum). This suggests that there is likely a regulatory factor in the serum involved in hypothalamic insulin BBB transport and receptor binding. Indeed, it is known serum factors such as triglycerides, free fatty acids and glucose can alter insulin BBB transport.^6^, ^11^

Previous studies have observed beneficial effects of acute RSG in cardiac ischemia/reperfusion models when administered 5 minutes prior to injury.^55^, ^56^ In brain endothelial cells, PPARβ and PPARδ are the predominant genes expressed compared to other members of the PPAR family.^57^ PPARγ is most abundantly expressed in adipose tissue compared to other metabolic organs. However, PPARγ is still expressed in endothelial cells and can regulate the release of nitric oxide ^58^ which has been shown to affect insulin BBB transport.^59^ Another study investigated the acute response of RSG treatment on endothelial function in healthy men and found that a single dose did not affect serum glucose or insulin levels 6 or 24 hours following treatment.^60^ Importantly though, endothelial function as measured by flow‐mediated endothelium‐dependent vasodilation was significantly increased following RSG. These data suggest a direct effect of RSG on the endothelium, independent of metabolic action, that can occur with a single administration in a short time period.

While RSG does not greatly penetrate the murine BBB (0.045% of an iv injected dose), uptake does occur rapidly, within 1 minutes.^28^ In addition, RSG is a substrate of p‐glycoprotein and, therefore, is readily transported out of the brain. However, we were interested in the direct effect that would be mediated at the luminal surface of the brain endothelial cell, rather than an effect within the CNS. Indeed, PPARγ activation is known to have a direct effect on cerebral vascular expression of proteins, including various adhesion molecules and metalloproteinases.^61^ Therefore, we know brain endothelial cells can respond directly to RSG and alter expression of proteins at the brain endothelial cell surface.

Finding methods to increase transport of insulin into the brain could elucidate potential pharmacologic solutions to conditions in which CNS insulin resistance occurs. An underlying question remains: What roles do current type 2 diabetes medications play in central insulin transport? Therefore, it is worth investigating whether RSG influences insulin BBB transport in a chronic insulin‐resistant state, both peripherally and centrally, in addition to how the ability of RSG to enhance insulin binding to brain endothelial cells might improve CNS insulin resistance. Our studies focus on a PPARγ agonist in determining the effect on insulin at the BBB, both regarding transport and binding. However, whether other type 2 diabetes medications have an acute or chronic impact on insulin BBB transport or binding remain to be determined.

## CONFLICTS OF INTEREST

The authors have no conflicts of interest to disclose.

## AUTHOR'S CONTRIBUTIONS

EMR conceived and designed the study. DCG and EMR planned and executed the experiments, analysed the data, interpreted the results and drafted the manuscript. WAB aided in data interpretation and critically reviewed the manuscript.

## ETHICS APPROVAL

The present study was designed and conducted in compliance with the Institutional Animal Care and Use Committee at the Veterans Affairs (VA) Puget Sound (Seattle, WA). The VA Puget Sound animal facility is certified by the Association for Assessment and Accreditation of Laboratory Animal Care International.

## Data Availability

The data that support the findings of this study are available from the corresponding author upon reasonable request.
